# Acquired neonatal bronchial stenosis after selective intubation: Successful managed with balloon dilatation

**DOI:** 10.1002/ccr3.2112

**Published:** 2019-03-29

**Authors:** Pierre Goussard, Julie Morrison, Adrie Bekker, Barend Fourie

**Affiliations:** ^1^ Faculty of Medicine and Health Sciences, Department of Paediatrics and Child Health Stellenbosch University and Tygerberg Hospital Cape Town South Africa

**Keywords:** balloon dilatation, bronchial stenosis, pulmonary interstitial emphysema, selective intubation, subglottic stenosis

## Abstract

Premature babies are prone to airway‐related complications. Selective intubation for the management of pulmonary interstitial emphysema may cause acquired bronchial stenosis. Balloon dilatation under fluoroscopy is a safe minimal invasive and successful intervention for acquired bronchial stenosis. Follow‐up bronchoscopy is needed due to risk of restenosis.

## 1 INTRODUCTION

Premature babies are prone to many complications, especially in the developing world where they have limited access to medical services, and the initiation of treatment is often delayed. Airway‐related complications are common, except for acquired subglottic stenosis. We report on a case of both subglottic and bronchial stenosis in a premature neonate following selective right main bronchus intubation and ventilation for the management of pulmonary interstitial emphysema (PIE). The usual treatment options for acquired bronchial stenosis would be either sleeve resection or stenting of the airway. Both the subglottic and the bronchial stenosis were successfully treated by balloon dilatation. The bronchial dilatation was done under fluoroscopy guidance. Acquired bronchial stenosis due to selective intubation has not been previously described. Balloon dilatation under fluoroscopy is a safe, minimally invasive, and successful intervention for acquired bronchial stenosis.

Premature babies are prone to many complications, especially in the developing world where they have limited access to medical services and often experience a delay in treatment initiation. Airway‐related complications are common, except for acquired subglottic stenosis.

Distal stenosis of the trachea and the main bronchi have been described in very small babies. The reported incidence of acquired trachea‐bronchial stenosis (ATBS) in mechanically ventilated babies ranges from 2%‐11%, though milder cases are often underdiagnosed.^1^

## 2 CASE PRESENTATION

A 28‐week‐old premature boy, with a birthweight of 1280 grams, was intubated with a 2.5 mm endotracheal tube via the nose and ventilated for severe hyaline membrane disease (HMD). After receiving two doses of surfactant, the premature neonate was successfully weaned off ventilation and extubated to nasal continuous positive airway pressure (CPAP). On day 9, his clinical course was complicated by a pulmonary hemorrhage, requiring re‐intubation. He was given another dose of surfactant and stabilized on high‐frequency oscillation ventilation (HFOV). The chest radiograph showed extensive bilateral pulmonary interstitial emphysema, with the left side more extensively involved in comparison to the right. A hemodynamically significant patent ductus arteriosus was treated by intravenous paracetamol. The baby's condition did not improve, and he was selectively intubated into his right main bronchus. The position of the endotracheal tube was radiologically confirmed, allowing the right lung to be oscillated while the left lung was rested. The baby was nursed on his left side for a period of 36 hours, after which the endotracheal tube was retracted into the trachea and secured in that position. The baby's ventilatory status subsequently improved, allowing for extubation on day 7 after the relapse. On day 28 of life, he presented with severe stridor, requiring re‐intubation. Difficulty during intubation suggested that subglottic stenosis might be present. One week after this re‐intubation a flexible bronchoscopy was performed, which revealed two major findings. The first was a Cotton grade 2 subglottic stenosis, and the other abnormality was near‐complete obstruction of the bronchus intermedius. The subglottic stenosis was dilated to 5 mm with the aid of a balloon dilator (Boston scientific Mustang™ balloon dilatation catheter). The bronchus intermedius was extremely narrow, and a 2.2 mm flexible bronchoscope was not able to pass through the area of stenosis.

**Figure 1:**
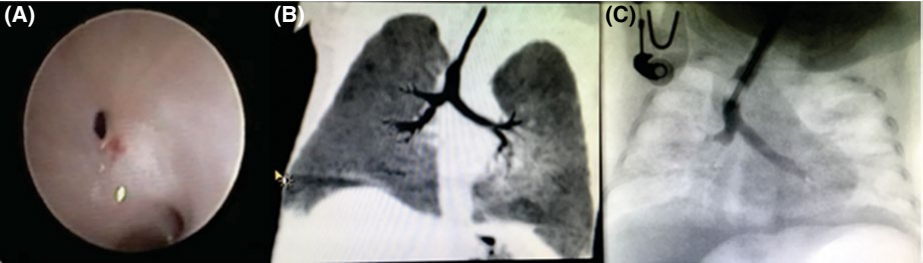
A, Bronchoscope image of the right main bronchus, demonstrating the pinhole size of the bronchus intermedius. B, CT‐scan image of the circumferential narrowing of bronchus intermedius just below the opening of the right upper lobe. C, Contrast study under fluoroscopy confirming the narrowing

After 2 weeks, the bronchoscopy was repeated and the subglottic region had improved to near normal in diameter. The bronchus intermedius stenosis, however, remained unchanged. A chest Computed Tomography (CT) scan was performed to determine the length of the bronchial stenosis. The CT scan confirmed that the stenosis involved a short segment and had a web like in configuration (Figure 1).

**Figure 2:**
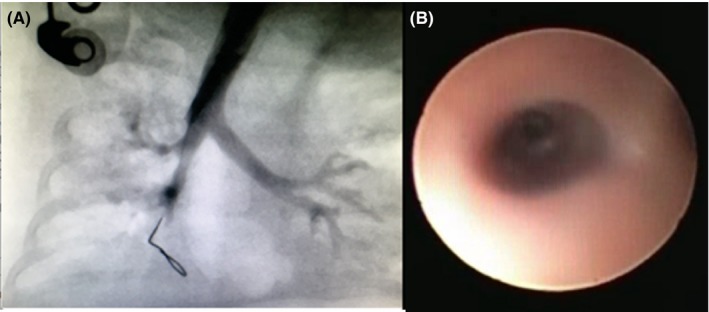
A, Post balloon dilatation of the bronchus demonstrating improvement in size of the bronchus. B, Bronchoscopy image 6 weeks after dilatation of the airway confirming a patent bronchus intermedius, which allows a 2.8 mm bronchoscope

It was decided to balloon dilate the stenosis under fluoroscopy. As the cardiac catheterization suite offered the best quality fluoroscopy, it was decided to perform the dilatation in the suite. The baby was intubated, and a guidewire was inserted into the right main bronchus under fluoroscopy. Water‐soluble contrast was injected, and the position of the airway identified. A 3.5 mm coronary artery balloon catheter was inserted into the area of stenosis via the guide wire, and the position was confirmed by fluoroscopy. The balloon was inflated at 16 atmospheric pressure for 20 seconds. This was repeated for another 20 seconds before water‐soluble contrast was reinjected, demonstrating significant decrease in the bronchial stenosis (Figure 2). Following the procedure, the baby was ventilated for less than 24 hours. At follow‐up bronchoscopy 2 weeks later, the stenosis had significantly improved, allowing a 2.8 mm flexible bronchoscope to pass comfortably through the stenotic region. The posterior part of the stenosis had completely resolved, with a small anterior shelf remaining. The baby was discharged with no known respiratory complications, and at follow‐up bronchoscopy 6 weeks, after the latter dilatation procedure, the airway remained patent and the baby remained asymptomatic, with a normal chest radiograph. Follow ‐up bronchoscopy was done due to the risk of restenosis and the fact that the baby was from a rural area, with limited medical services.

## 3 DISCUSSION

Pulmonary interstitial emphysema is a relatively common manifestation of pulmonary baro‐trauma related to mechanical ventilation.^2^ The incidence of this phenomenon has been on the decline, due to the use of surfactant and permissive hypercapnia.

Various modalities for the treatment of PIE have been used, including position therapy, HFOV, visceral pleurotomy, and selective bronchial intubation.^2^

For selective intubation to be successful, the tip of the endotracheal tube (ETT) must be passed into the opening of the right main bronchus and kept in that position to facilitate collapse of the left lung. The duration of this procedure depends on how quickly the lung collapses and if the baby can tolerate single lung ventilation.The preferred duration of selective main stem intubation reported in the literature varies arbitrarily from 1 to 10 days.^2^, ^3^ In some cases, the combination of selective intubation and HFOV have been used with an approach of low oscillatory frequency and a reduced mean airway pressure.^3^ This is important in the management of PIE as this decreases the driving pressure of the gas through the leak site.

The pathogenesis of ATBS is unknown, but one of the possible factors that reportedly plays a role in the development of the condition includes deep endotracheal suctioning. This has previously been confirmed by Bayley et al in rabbit studies.^4^ Other possibilities include movement of the endotracheal tube during the flexion or extension of the head as this may induce ulceration of the mucosa/tracheal wall at the tip of the tube. These actions may lead to the introduction of inflammation, followed by scarring, which could result in stenosis. Other factors are insufficient humidifying of the inhaled gasses and repeated intubation. The role of HFOV in ATBS in small premature neonates have not been described, however, if the baby is intubated into his right main bronchus, which has a smaller diameter than the trachea, and the endotracheal tube is lodged in the lumen, as is required for selective intubation, the constant movement of the tube at high speed may result in ulceration, albeit purely speculative.

The options for the treatment of acquired bronchial stenosis would be either sleeve resection or stenting of the airway. Both these procedures are very invasive. A stent placement, although not technically difficult, can be very problematic due to the anatomical location of the stenosis and the complications resulting from granulation tissue. Placing a stent in the right main bronchus may cause obstruction of the upper lobe bronchus and protrusion of the stent into the trachea. Biodegradable stents are a better option, but they are very expensive and not freely available.

Previously airway dilatations in babies have only been described using a rigid scope and a coronary artery balloon.^5^

## 4 CONCLUSION

Extremely premature babies are vulnerable to many complications. Acquired bronchial stenosis due to selective intubation has not been previously described. Balloon dilatation under fluoroscopy is a safe, minimally invasive, and successful intervention for acquired bronchial stenosis.

Consent was given by the parents of the infant for publication of the case.

## CONFLICT OF INTEREST

None declared.

## AUTHOR CONTRIBUTION

Pierre Goussard: is a pediatric pulmonologist and is involved with the treatment of the patient and writing the manuscript. Adrie Bekker: is a neonatologist and responsible for the neonatal management of the baby and editing the manuscript. Julie Morrison: is a pediatric pulmonologist and is responsible for the management of baby and writing the manuscript. Barend Fourie: is a pediatric cardiologist and is responsible for the catheterization of the baby and writing the manuscript.
